# Long COVID: Costs for the German economy and health care and pension system

**DOI:** 10.1186/s12913-023-09601-6

**Published:** 2023-06-14

**Authors:** Afschin Gandjour

**Affiliations:** Frankfurt School of Finance & Management, Adickesallee 32-34, 60322 Frankfurt, Germany

## Abstract

**Background:**

Patients with acute COVID-19 can develop persistent symptoms (long/post COVID-19 syndrome). This study aimed to project the economic, health care, and pension costs due to long/post-COVID-19 syndrome with new onset in Germany in 2021.

**Methods:**

Using secondary data, economic costs were calculated based on wage rates and the loss of gross value-added. Pension payments were determined based on the incidence, duration, and amount of disability pensions. Health care expenditure was calculated based on rehabilitation expenses.

**Results:**

The analysis estimated a production loss of 3.4 billion euros. The gross value-added loss was calculated to be 5.7 billion euros. The estimated financial burden on the health care and pension systems due to SARS-CoV-2 infection was approximately 1.7 billion euros. Approximately 0.4 percent of employees are projected to be wholly or partially withdrawn from the labor market in the medium term due to long/post-COVID with new onset in 2021.

**Conclusion:**

Costs of long/post-COVID-19 syndrome with new onset in 2021 are not negligible for the German economy and health care and pension systems but may still be manageable.

## Introduction

The British National Institute for Health and Care Excellence [33] defines “long COVID” as health problems that occur during or after the acute phase of a SARS-CoV-2 infection and persist for more than 4 weeks. Post-COVID-19 syndrome is the term used to describe symptoms that are still present more than 12 weeks after the start of the SARS-CoV-2 infection and cannot be explained otherwise [33]. The leading symptoms of many post-COVID sufferers are considerable exhaustion (fatigue) and limited resilience [20]. The possible long-term consequences of a SARS-CoV-2 infection also include permanent damage to the lungs and the associated deterioration in lung function, impairment of kidney function, cardiovascular diseases (e.g., myocarditis, heart attack, stroke, and thromboembolism), diabetes mellitus, and psychological problems, and [40]. Risk factors include obesity and female gender [48]. Although post-COVID can also affect people who have had a mild course, those who have had a severe course and thus had to be hospitalized are at a higher risk of suffering long-term consequences. A study from England shows that over 70 percent of all hospitalized COVID-19 cases have not recovered even after 12 months [38]. After a mild infection, around 10 percent of those affected meet the post-COVID criteria ([20] published in May 2022). It should be noted that twice-vaccinated people with breakthrough infection had a significantly lower probability of developing long/post-COVID than unvaccinated people [1, 2, 39]. Similarly, those who had been boosted had a lower risk of post-COVID than those twice vaccinated [44]. According to the Federal Government's Expert Council on COVID-19 [20], "[t]he effects of these potential long-term complications on society and the social security and health system (…) are of great importance to society as a whole given the high number of infections" [author’s translation]. Therefore, this study estimated the costs of long/post-COVID cases with new onset in 2021 for the German economy (measured in terms of loss of production and gross value added (GVA)) and the German health care and pension system. The underlying purpose was to identify a potential need for policymaking, such as investments in research into long/post-COVID and new therapeutic approaches.

## Methods

This study uses secondary data from various sources such as statutory insurance (including sickness funds) and federal institutions. Production loss due to post-COVID was determined using wage rates according to the methodology of the Federal Institute for Occupational Safety and Health [6]. Economic costs were calculated based on the GVA loss [6]. GVA is a measure of the value of goods and services produced in an area, industry, or sector of an economy [55].

The proportion of COVID-19 patients employed before infection who underwent rehabilitation due to long/post-COVID was calculated by dividing the number of COVID-19 rehabilitations for 2021 by the estimated number of COVID-19 patients employed before infection. The calculation considered the average lead time of COVID-19 (i.e., the duration between infection and rehabilitation) and the national employment rate (75.5 percent based on [47]).

Pension payments triggered by rehabilitation in 2021 were estimated based on the duration and the average amount of disability pensions. The proportion of COVID-19 patients who received a partial or full disability pension was projected based on the estimated success rate of disability prevention after rehabilitation.

The costs to the German healthcare system due to long/post-COVID were determined based on rehabilitation expenses incurred by the German statutory pension insurance, social accident insurance, and statutory health insurance (SHI). The SHI covers the rehabilitation of retired people and family members. However, the number of rehabilitations due to long/post-COVID covered by the SHI could not be directly retrieved because of the absence of diagnostic codes in the dataset [9]. Therefore, rehabilitations of retired patients were projected based on the proportion of retired patients receiving respiratory rehabilitation 6 months after treatment with extracorporeal membrane oxygenation (ECMO) on the intensive care unit due to COVID-19. This calculation does not consider rehabilitations of retired patients with long/post-COVID who were not admitted to the intensive care unit.

Since no approved therapy was available for long/post-COVID available and the therapy was based on symptoms [14], no significant expenditure on diagnostics, physiotherapy, and other treatments was expected in addition to rehabilitation expenditure.

## Results

The proportion of COVID-19 patients on sick leave who were unable to work for at least 12 weeks owing to SARS-CoV-2 infection in 2020 was approximately 10 percent according to an evaluation of data from the second-largest sickness fund in Germany (Barmer [3, 4]). If 84 days of absenteeism per post-COVID patient and a production loss of 124 euros per day due to incapacity to work (full-time equivalent) (BAuA [6]) were assumed, a production loss of 10,416 euros per post-COVID patient would be calculated. The number of new-onset post-COVID-19 patients employed before infection in 2021 was approximately 240,000 based on incidence data from the Robert Koch Institute ([40]) and  after the conversion of the number of employed people into full-time equivalents [26]. This results in a production loss of 3.4 billion euros (Table 1). However, the costs are underestimated because the period of incapacity to work during post-COVID is longer than 12 weeks on average and production loss also occurs due to presenteeism. Assuming that productivity loss due to presenteeism is approximately 70 percent of the productivity loss due to absenteeism [31], the production loss is 5.9 billion euros. In addition, an employee's absence from work can have a negative impact on the productivity of other employees in the company, such as team work [34]. On the other hand, production loss is overestimated because post-COVID occurs more frequently in women [48] and the female gender is associated with lower income and thus a smaller production loss [57]. In addition, some lost productivity is partially compensated for by other employees and some positions can be refilled. Regarding the latter point, the vacancy period, that is, the time between the desired appointment date and cancellation of the vacancy was less than three months for 52 percent of the reported positions in March 2022 [5] and thus was below the defined minimum post-COVID period.

**Table 1:** Economic, health care, and pension costs due to long/post-COVID-19 syndrome in Germany

	Unit costs	Number of units	Total costs
Production loss	€124 per day	27,610,261 days	€3,423,672,399
Loss of gross value	€205 per day	27,610,261 days	€5,660,103,562
Rehabilitation costs	€3000 per stay	110,704 stays	€332,111,250
Pension payments	€109,024 to €169,344 per case	17,141 cases	€2,067,814,765

Assuming a loss of GVA per day of incapacity to work of 205 euros (full-time equivalent) [6], a loss of GVA of 17,220 euros per post-COVID patient was calculated. At the federal level, there was a loss of GVA of 5.7 billion euros or 0.2 percent of the gross domestic product (Table 1). However, this calculation only includes the direct effect on the economy or value creation. Indirect added value effects through the purchase of inputs from other sectors and the induced added value through the re-spending of the directly and indirectly generated income are not considered [25]. In addition, overestimation or underestimation of the loss of GVA can result from the factors mentioned in the previous paragraph for the projection of productivity loss.

When calculating the costs of the pension system, it must be considered that for COVID-19 patients the average time between infection and the start of rehabilitation was 15.27 weeks [7]. Considering the above sickness fund data [3], the proportion of COVID-19 patients potentially eligible for rehabilitation was therefore less than 10 percent. In fact, the German statutory pension insurance [19] reported only approximately 10,000 COVID-19 rehabilitations (inpatients) in 2021. In addition, there are an estimated 100,000 rehabilitants (inpatients and outpatients) for whom statutory accident insurance recognized COVID-19 as an occupational disease in 2021 [16]. Dividing the total number of rehabilitations (approximately 110,000) by the number of COVID-19 patients employed before infection yields the proportion of COVID-19 patients employed before infection who undergo rehabilitation (2.3 percent). Considering the average rate of successful disability prevention after rehabilitation [8, 17], the proportion of COVID-19 patients who receive a partial or full disability pension is estimated to be 0.4 percent (i.e., almost every 300^th^ COVID-19 patient and every 30^th^ post-COVID patient).

Since the average age of patients with long/post-COVID and incapacity to work was 49 years [3], the disability pension was paid over an average period of approximately 16 years (until the regular old-age pension occurs). The pension payments triggered by rehabilitation in 2021, considering the average amount of disability pension [15, 18], were estimated to be around 2.1 billion euros (Table 1).

However, the financial burden on the German healthcare system due to long/post-COVID was likely lower. With an expenditure of approximately 3,000 euros per COVID rehabilitation patient [32], the total expenditure incurred by the German statutory pension insurance and German social accident insurance for rehabilitation was around 332 million euros in 2021.

To project the rehabilitation expenditure incurred by the SHI, the number of patients older than 60 years who survived after treatment with ECMO due to COVID-19 until September 2021 in Germany (*n* = 375 based on [22]) was multiplied by the proportion of survivors receiving respiratory rehabilitation 6 months after discharge in a European cohort (17 percent based on [29]). The resulting expenditure was approximately 190,000 euros, thus contributing little to the total rehabilitation expenditure (Table 1).

## Discussion

The analysis shows that long/post-COVID produces costs particularly in terms of productivity loss or GVA and thus for the national economy. The estimated loss of GVA (5.7 billion euros) was in the range of a projection for the decline in gross domestic product in Great Britain because of health consequences related to the pandemic [50]. The estimate of 9.3 billion euros (8 billion pounds) for 2022 not only took into account a reduction in the number of workers of up to 200,000 due to long-term illness, such as late effects of COVID-19, but also a further reduction in workers in the same amount due to “unspecific” pandemic factors [50]. Similar to the present study, the British estimate is based on the linear relationship between the number of lost workers and economic growth (see Table C.2. in the underlying study by the British Office for Budget Responsibility [35]). As a word of caution, under perfect competition in the labor market, the marginal product of a worker corresponds to the real wage [54]. Furthermore, the marginal product of labor eventually falls [53]. Therefore, the economic costs caused by long/post-COVID are best approximated by wages, not GVA. An approximation using GVA is valid under the assumption of a constant marginal product [12].

Conversely, the socio-economic burden on the German health care and pension system due to long/post-COVID with new onset in 2021 does not appear to be dramatic. Nevertheless, this finding is subject to uncertainty in input parameters, for example, non-rehabilitation expenditure for statutory health insurance and the representativeness of the Barmer sickness fund data on the proportion of COVID-19 patients on sick leave over 12 weeks (the Barmer covers 8.7 million lives and thus only 10 percent of the German population). The assumption that no significant expenditure on diagnostics, physiotherapy and other treatments was expected in addition to rehabilitation expenditure was supported by a survey of long-COVID patients publicly insured by the sickness fund IKK Südwest [27], in which only one third of those surveyed stated that they used medical services. Nevertheless, a detailed analysis of  the inpatient expenditure of all hospitalized long/post-COVID patients in Germany in 2021 yielded a cost of 137 million euros [51]. However, the study did not exclude long/post-COVID patients in whom the reason for being admitted to the hospital was not caused by SARS-CoV-2 infection but was present before infection, thus leading to a potential overestimation of costs. Studies conducted abroad yielded similar results. A British model calculated disease-specific costs of 180 pounds per patient with long/post-COVID [41]. A US cohort study also showed only 0.7 additional physician visits and additional costs of 224 dollars (211 euros) over six months after COVID-19 diagnosis compared to the period before diagnosis [28]. As a word of caution, the calculation considered not only patients with long/post-COVID but also COVID-19 patients without persisting symptoms. This reduced the average cost.

Notably, rehabilitation and training concepts for post-COVID have not yet been optimized [38], which may reduce the success of current rehabilitation programs. In the future, improvements in medical treatment standards for rehabilitation could lead to a decline in disability pensions (currently, this is controversial [10]).

Long/post-COVID as a consequence of infection during the Omicron wave was not considered in this analysis, as it could only be recorded in 2022 (the first cases of infections with the Omicron variant were reported in Germany at the end of November 2021). Data from the sickness fund AOK showed that the proportion of employees who were on sick leave due to long/post-COVID from February to April 2022 (i.e., during the pandemic wave dominated by the Omicron variant) was lower than in the phase in which the Delta variant dominated [56]. However, the extent to which the proportion of COVID-19 patients on sick leave who then receive rehabilitation or disability pensions can be transferred to the Omicron wave is still unclear. In the UK, the proportion of patients with self-reported long-term COVID-19 symptoms increased from 1.3 million at the turn of the year 2021/2022 to 2 million at the beginning of May 2022 [36]. This increase was likely associated with Omicron variant infections. That put around one-third of the UK’s long-COVID cases down to the Omicron variant. In terms of future long/post-COVID cases, it is somewhat reassuring that recently published data from the UK COVID-19 Infection Survey [37] indicate that reinfections decrease the odds of new-onset, self-reported long COVID by 28 percent.

The similarities between long/post-COVID and Myalgic Encephalomyelitis/Chronic Fatigue Syndrome (ME/CSF) have been investigated. ME/CSF, the cause of which is still unknown, is also diagnosed in a subgroup of long-COVID patients six months after infection [13]. Estimates of the per capita costs for patients with ME/CSF are generally significantly higher than those for long/post-COVID. However, studies on the costs of ME/CSF are limited if they do not calculate disease-specific costs (e.g., [11]); determining disease-specific costs requires subtracting costs from a matched control group. Furthermore, foreign surveys on ME/CSF also found a high number of self-reported doctor consultations after the diagnosis of the disease ([11], which seems to contradict the above-mentioned findings for long/post-COVID in Germany [27].

Two controlled clinical trials testing drugs (RSLV-132 and TNX-102 SL) against long COVID have completed enrollment (ClinicalTrials.gov, searched on May 1, 2023). Varying symptoms among patients, and the absence of biomarkers require a trial-and-error approach that extends the clinical testing phase and delays market approval [43]. Nevertheless, a randomized, double-blind, placebo-controlled clinical trial investigating AXA1125 demonstrated a “marked symptomatic improvement” [21]. The severity of long/post-COVID and the lack of therapeutic alternatives are likely to harbor a certain price potential that could significantly affect future long/post-COVID spending. However, savings from avoided production losses or health care and pension expenses must also be considered. The price of Paxlovid (nirmatrelvir/ritonavir), which has been approved in the European Union since January 28, 2022, for the treatment of symptomatic, non-hospitalized COVID-19 patients with a high risk of a severe course of the disease, could provide a pricing benchmark. In the pivotal study, Paxlovid significantly reduced the likelihood of hospitalization or death [24]. Observational data suggest that Paxlovid reduces post-COVID risk by 26 percent in patients with at least one risk factor for progression to severe acute COVID-19 illness [58]. In the USA, the price is approximately 530 dollars (509 euros) per patient [49]. However, the market volume of a new drug against long COVID is likely to significantly exceed that of Paxlovid in its current label since the approved patient population and thus the number of potential customers are also significantly larger due to the relatively high probability of long/post-COVID in non-risk patients and vaccinated patients. In addition, the market volume should not be limited in time, as is the case with Paxlovid, where many patients have developed resistance to Paxlovid, and thus rendered Paxlovid ineffective [45].

In connection with the previously unprescribed packs of Paxlovid in Germany [52], the fundamental question arises as to what extent the costs for ordered but unused packs of a new drug should be added to the social costs of long/post-COVID. A million Paxlovid packs were purchased by the Federal Ministry of Health at an undisclosed price. Applying the US price, the order costs for Paxlovid were around half a billion euros.

A reduction in labor supply due to long/post-COVID could exacerbate the shortage of skilled workers, which will increase demographically in the coming years. The increase in depressive disorders that occurred during the pandemic [46] as a direct consequence of SARS-CoV-2 infections and a symptom of long/post-COVID [42] contributes to this. This study suggests that an estimated 0.4 percent of employees will be wholly or partially withdrawn from the labor market in the medium term, would the result of this analysis for 2021 (the proportion of SARS-CoV-2 infections that result in a disability pension) also apply to the following years. The underlying assumption is that every employee will be infected with SARS-CoV-2 in the medium term. A US study [23] reached a similar conclusion as early as June 2022 regarding the reduction in labor supply due to long/post-COVID (0.2 percent). Similarly, a British analysis showed that between 2019 and 2021 the proportion of employees unable to work increased by 0.4 percent and attributed this increase to the pandemic [39]. Based on the UK Understanding Society COVID-19 Survey, the same study concluded that 3.7 percent of long/post-COVID patients were unavailable to work due to their infection. This corresponds almost exactly to the proportion of patients with a disability pension in the analysis. At the same time, some forecasts predict an increase in the need for healthcare professionals to care for patients with long/post-COVID [30]. However, long/post-COVID could also serve as a catalyst for changes in the working world, such as more flexible working hours [59].

From the perspective of the economy, a balance must be struck between the interest in coronavirus protection measures, which limit the occurrence of long/post-COVID, also caused by SARS-CoV-2 reinfections, and the resulting reduction in labor supply on one hand and unrestricted economic activities on the other hand. The trade-off between the loss of GVA due to long/post-COVID due to absenteeism and presenteeism on one hand and the loss of GVA through protective measures on the other is similar.

## Conclusions

The analysis shows that long/post-COVID produces costs, particularly in terms of productivity loss and GVA, and consequently for the national economy. Conversely, the socio-economic burden on the German health care and pension system due to long/post-COVID with new onset in 2021 does not appear to be dramatic. Given that 0.4 percent of employees are projected to be wholly or partially withdrawn from the labor market in the medium term due to long/post-COVID, it appears crucial to monitor incident cases from 2022 on to inform policymakers about the growing size of the problem.

## Data Availability

This study is a modelling study and uses secondary data from various sources. The data sources are mentioned in the text and are included the reference list. They are publicly available.
